# Development and validation of long-acting recombinant human TSH using SAFA technology

**DOI:** 10.1530/ERC-24-0284

**Published:** 2025-02-17

**Authors:** Daham Kim, Min Jeong Kang, So Jeong Lee, Yoon Hee Cho, Gunuk Zi, Jeongsuk An, Jinjoo Park, Jaekyu Han, Susan Chi, Sang-hoon Cha, Eun Jig Lee

**Affiliations:** ^1^ Department of Internal Medicine, Institute of Endocrine Research, Yonsei University College of Medicine, Seoul, South Korea; ^2^ Department of Tropical Medicine, Yonsei University College of Medicine, Seoul, South Korea; ^3^ AprilBio Co., Ltd, Biomedical Science Building, Kangwon National University, Chuncheon, South Korea

**Keywords:** neonatal Fc receptor, radioiodine therapy, recombinant proteins, thyroid neoplasms, thyrotropin

## Abstract

Thyrogen, a recombinant human thyroid-stimulating hormone (rhTSH), has a short half-life in the bloodstream, which necessitates multiple doses during treatment. Therefore, we developed a new long-acting rhTSH using anti-serum albumin Fab-associated (SAFA) technology to validate its biological activity through *in vitro* assays, pharmacokinetic studies in healthy mice and pharmacodynamics studies in a thyroid-stimulating hormone (TSH)-suppressed mouse model. SAFA-TSH was produced using a Chinese hamster ovary expression system. To verify its biological activity, we generated Nthy-ori 3-1 cells stably overexpressing TSHR and measured the production of cyclic adenosine monophosphate (cAMP). In a rat study, slow-release triiodothyronine (T3) pellets were implanted 3 days before administering Thyrogen or SAFA-TSH to measure the amount of thyroxine (T4) release alone resulting from exogenous administration. SAFA-TSH increased cAMP production dose-dependently, but less effectively than Thyrogen at similar concentrations. SAFA-TSH required six times the dose of Thyrogen to achieve similar cAMP levels, likely due to differences in molecular weight and relative bioactivity. In a rat study, SAFA-TSH produced elevated thyroid hormone levels well after the decline in the response to Thyrogen. SAFA-TSH had significantly higher cumulative effects on T4 and free T4 levels compared with Thyrogen, as observed by a more than two-fold higher average area under the effect curve of 262.56 vs 118.89 μg × h/dL and 127.47 vs 60.75 μg × h/dL, respectively. SAFA technology created successful long-acting TSH that demonstrated bioactivity. These findings endorse the continued development of SAFA-TSH for clinical use, highlighting its potential as a significant advancement treating thyroid cancer patients.

## Introduction

Thyroid cancer is the most common endocrine malignancy and differentiated thyroid cancer (DTC) accounts for >90% of all thyroid cancers (Choi *et al.* 2022, Kitahara & Schneider 2022). The primary treatment for patients with DTC is surgery, followed by radioactive iodine (RAI) therapy, when indicated, and thyroid hormone suppression therapy (Lee *et al.* 2024*a*,*b*, Ryu *et al.* 2024). The goals of RAI therapy in DTC are as follows: remnant ablation, adjuvant treatment or treatment of known disease (Borges de Souza & McCabe 2021, Pacini *et al.* 2022). Thyroid-stimulating hormone (TSH) stimulates the uptake of radioiodine by thyroid tissue (Hasbek & Turgut 2016). This can be achieved through two methods: either thyroid hormone withdrawal (THW) or administering recombinant human TSH (rhTSH).

Many patients experience acute short-term hypothyroid symptoms induced by THW, which significantly decrease their quality of life (Lim *et al.* 2015). Thyrogen, a rhTSH, was introduced in 1999 to elevate TSH levels without requiring THW (Haugen *et al.* 1999, Pacini *et al.* 2006). It is administered via buttock injection for two consecutive days. Thyrogen is used in DTC for diagnosis and thyroid remnant ablation; however, it is not approved for distant metastases (Haugen *et al.* 2016, Pacini *et al.* 2022). Thyrogen stimulates lower thyroglobulin levels than THW and may not maximize short-term iodine uptake, potentially reducing efficacy (Haugen *et al.* 1999, Kowalska *et al.* 2015). Recent studies have shown that in patients with distant metastases, there were no significant differences in the disease control rate between the rhTSH group and the THW group; however, the evidence remains insufficient (Simoes-Pereira *et al.* 2021, Park *et al.* 2024). Due to its short half-life, requiring a multidose regimen, and its potential inability to adequately stimulate thyroid cancer cells, there is a need for the development of long-acting TSH formulations (Park *et al.* 2013). These may significantly enhance treatment efficacy, improve patient convenience and provider flexibility and advance treatment and diagnosis.

Anti-serum albumin Fab-associated (SAFA) technology extends the serum half-life of protein therapeutics and utilizes human anti-serum albumin Fab to achieve a prolonged kinetic profile through the neonatal Fc receptor recycling mechanism (Kang *et al.* 2016, Kang *et al.* 2017, Cho *et al.* 2020). Using this technology, long-acting versions of recombinant human interferon beta, feline granulocyte colony-stimulating factor, human follicle-stimulating hormone and human IL-18BP have been developed (Ji *et al.* 2019, Chi *et al.* 2021, Jang *et al.* 2023, Kim *et al.* 2023). In the phase 1 trial of APB-R3 (SAFA-IL-18BP) (NCT05715736), the drug’s mean elimination half-life ranged from 222.28 to 342.28 h, and its mean residence time (MRT) ranged from 318.48 to 402.89 h. Importantly, it was found to be safe and well-tolerated. These findings underscore the potential of SAFA technology as a long-acting therapeutic option in clinical scenarios.

Therefore, this study aimed to develop a new long-acting rhTSH using SAFA technology to validate its biological activity through *in vitro* assays, pharmacokinetic (PK) studies in healthy mice and pharmacodynamics (PD) studies in a TSH-suppressed mouse model.

## Materials and methods

### SAFA-TSH production

SAFA-TSH was produced by chinese hamster ovary (CHO) glutamine synthetase null−/− K1 cell (Horizon Discovery, UK) and purified as per previously established methods by AprilBio Co., Ltd (Republic of Korea) (Ji *et al.* 2019, Kim *et al.* 2023). SAFA-TSH v1 (SAFA heavy-chain – TSH beta subunit and SAFA light-chain – TSH alpha subunit) and SAFA-TSH v2 (SAFA heavy-chain – TSH alpha subunit and SAFA light-chain – TSH beta subunit) were created and evaluated for expression efficiency. SAFA-TSH with ≥95% purity was used for all experiments.

### Cell culture

The human normal thyroid epithelial cell line Nthy-ori 3-1 was purchased from the European Collection of Cell Culture (ECACC, UK). Cells were cultured in RPMI-1640 medium (Gibco, Thermo Fisher Scientific, USA), supplemented with 10% fetal bovine serum (HyClone, USA) and 1% penicillin/streptomycin (Hyclone) in a humidified atmosphere of 5% carbon dioxide and 95% air at 37°C.

### Stable cell line generation

First, the human TSH receptor (TSHR) gene was inserted into the lentiviral vector pLECE3-green fluorescent protein (GFP) to generate pLECE3-hTSHR-GFP. Lentiviral particles were generated and purified for infection of Nthy-ori 3-1 cells. Next, GFP-positive single cells were sorted into a 96-well plate, as previously described (Kim *et al.* 2023). Through the expansion of single-cell clones and functional assays, the most promising cell clones were selected for further experiments.

### Measurement of cyclic AMP production

Cyclic adenosine monophosphate (cAMP) production was conducted as previously described (Kim *et al.* 2023). Nthy-ori 3-1 and Nthy-ori 3-1_TSHR cells were seeded (1.0 × 10^6^ cells/plate) and cultured in 6 cm plates. The next day, after starvation for 4 h, the cells were stimulated with Thyrogen (Genzyme Therapeutics, USA) or SAFA-TSH at different concentrations in 0.5 mM isobutylmethylxanthine (Sigma-Aldrich, USA) for 15 min at 37°C. cAMP concentrations were measured using a cAMP XP assay kit (Cell Signaling Technology, USA), with each experiment including a standard curve for calculation.

### Animal experimental design

The Institutional Animal Care and Use Committees of Yonsei University Health System in Seoul, South Korea, approved all animal experiments performed in this study (approval no. 2022-0159). Male Sprague–Dawley (SD) rats were obtained from Orient Bio (South Korea) and kept in controlled conditions (22°C, 12  h light:12  h darkness cycle) with access to rodent chow and tap water. The rats were allowed to acclimate for 1 week before starting the study.

For the PK study, Thyrogen 0.5 mg/kg, SAFA-TSH 1.5 mg/kg or SAFA-TSH 4.5 mg/kg were injected subcutaneously into 6-week-old male SD rats (*n* = 5 in each group). Blood was collected through the jugular vein, with 0.3 mL whole blood drawn and injected into a Vacutainer tube with a clot activator. Sampling was done ten times: before (0 h) and after administration at 0.5, 1, 2, 4 and 8 h and on days 1, 2, 3 and 4. The blood was coagulated at room temperature for 20–30 min, centrifuged at 1,500 ***g*** for 10 min and the serum was stored in tubes at −70°C. The serum concentrations of Thyrogen and SAFA-TSH were determined by quantitative enzyme-linked immunosorbent assay (ELISA). Briefly, after coating with anti-follicle-stimulating hormone (FSH) alpha antibodies (Gentaur, Belgium), Thyrogen or SAFA-TSH was bound and detected with horseradish peroxidase (HRP)-conjugated anti-TSH beta antibodies (Novus Biologicals, USA). The PK parameters were evaluated using a Phoenix® WinNonlin® (ver. 8.3, Certara, USA). Meanwhile, the PK study was performed by HLB bioStep Co., Ltd (South Korea).

For the PD study, 6-week-old male SD rats were randomly divided into three groups (*n* = 6–8 in each group). All rats, including those in the control group, were anesthetized with isoflurane, and a 0.5 mg triiodothyronine (T3) pellet (Innovative Research of America, USA) was implanted subcutaneously. After 3 days, a single dose of Thyrogen (0.4 mg/kg) or SAFA-TSH (2.4 mg/kg) was injected subcutaneously. The dosage ratio of Thyrogen to SAFA-TSH was determined based on the results of the *in vitro* functional assays conducted in this study. The control group was injected with saline instead of drugs. Blood was obtained from the tail vein at several time points: before administration (0 h), 6 h post-administration and on days 1, 2, 3, 4, 5 and 7 thereafter. Blood samples were processed for serum to analyze thyroxine (T4) and free T4 levels, measured by Seoul Clinical Laboratories (SCL, South Korea) using an electrochemiluminescence assay (ECLIA, Roche Diagnostics, USA).

### Statistical analysis

A IBM SPSS statistics version 25.0 (IBM, USA) was used for all statistical analyses. Data are presented as the means ± standard errors of the mean. Statistical significance was determined using the Mann–Whitney U test. Differences were considered statistically significant at a *P*-value of <0.05.

## Results

### SAFA-TSH characterization

**Figure 1:**
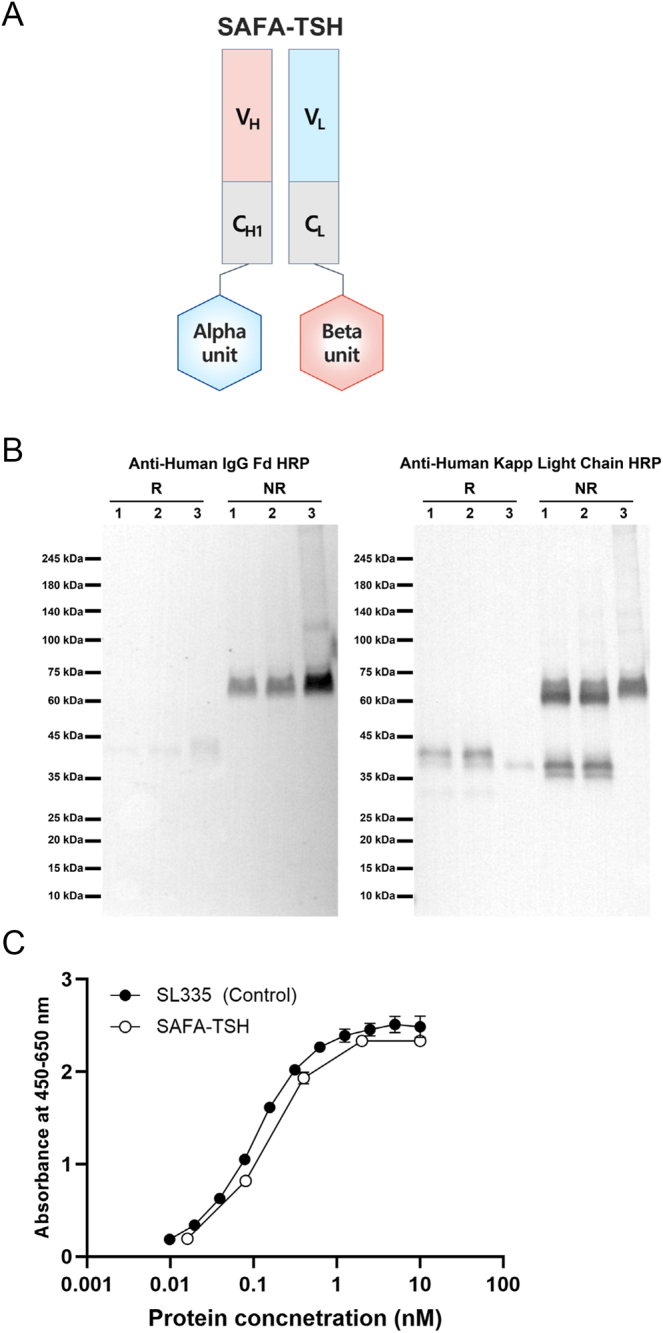
The characteristics of anti-serum albumin Fab-associated (SAFA)-TSH. (A) Illustration of SAFA-TSH, (B) western-blotting with anti-human IgG Fd horseradish peroxidase (HRP) (left) or anti-human kappa light-chain HRP (right) antibody under non-reducing (NR) or reducing (R) conditions. (1) SAFA-TSH v1 (old), (2) SAFA-TSH v1 (new), (3) SAFA-TSH v2, (C) Human serum albumin binding capacity of SAFA-TSH. A full color version of this figure available at https:// doi.org/10.1530/ERC-24-0284.

The illustration of SAFA-TSH is depicted in Fig. 1A. Initially, SAFA-TSH v1, where the SAFA heavy-chain was linked to the TSH beta subunit and the light-chain to the TSH alpha subunit, exhibited poor expression of the heavy-chain and inadequate structure formation under non-reducing conditions. Consequently, SAFA-TSH v2 was developed, with the heavy-chain linked to the TSH alpha subunit and the light-chain to the TSH beta subunit, showing improved expression efficiency in CHO cells compared with v1 (Fig. 1B). Consequently, all subsequent experiments were carried out using SAFA-TSH v2. In addition, the binding affinity of SAFA-TSH to human serum albumin was found to be comparable to SL335 (SAFA body), as shown in Fig. 1C.

### *In vitro* functional assays

**Figure 2:**
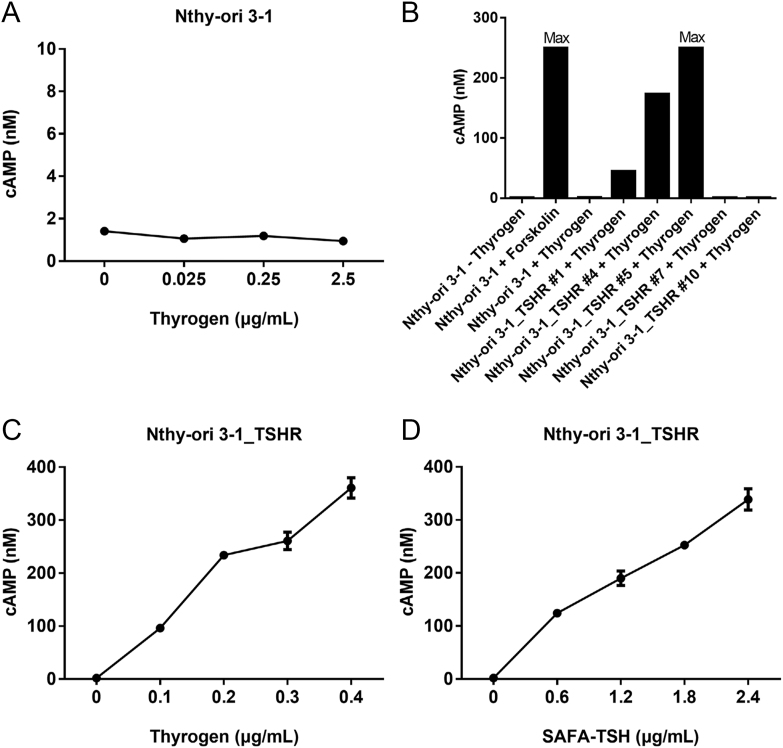
cAMP production for Thyrogen or anti-serum albumin Fab-associated (SAFA)-TSH *in vitro*. (A) cAMP production of Nthy-ori 3-1 cells treated with Thyrogen at indicated dose, (B) cAMP production in generated Nthy-ori 3-1 cell clones overexpressing TSH receptor (TSHR) treated with Thyrogen at 2.5 μg/mL, (C and D) cAMP production of Nthy-ori 3-1 cells treated with Thyrogen or SAFA-TSH at indicated doses. Line plots indicate the mean ± standard error of the mean (SEM).

Nthy-ori 3-1 cells initially showed no increase in intracellular cAMP levels when exposed to varying doses of Thyrogen (Fig. 2A). Hence, subsequent experiments involving TSH treatment were deemed impractical. Consequently, Nthy-ori 3-1_TSHR cells were created by lentiviral overexpression of the TSHR gene. These cells, treated with Thyrogen at 2.5 μg/mL, demonstrated measurable cAMP production (Fig. 2B), with Nthy-ori 3-1_TSHR #5 identified as the most promising cell clone for further studies.

**Figure 3:**
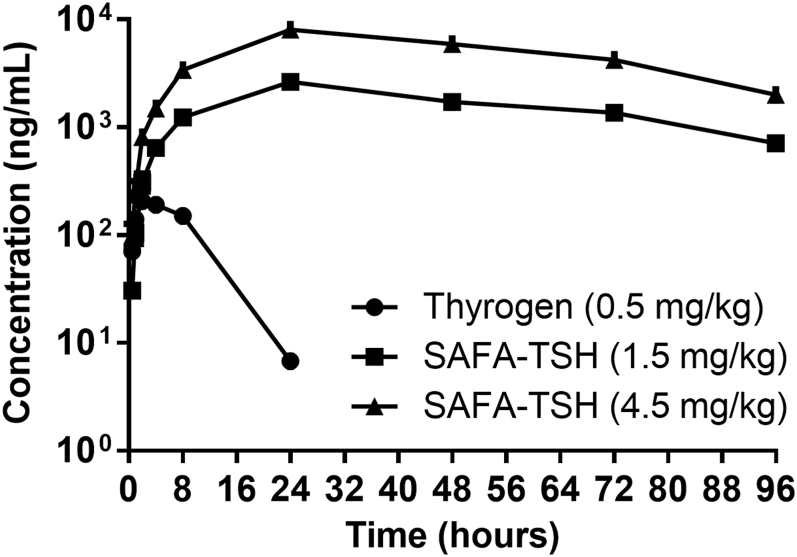
PK study results of anti-serum albumin Fab-associated (SAFA)-TSH. Line plots indicate the mean ± standard error of the mean (SEM).

The ability of SAFA-TSH to activate the TSHR was evaluated in Nthy-ori 3-1_TSHR cells using different concentrations of Thyrogen and SAFA-TSH (Fig. 3C and D). While both compounds increased cAMP levels in a dose-dependent manner, SAFA-TSH exhibited lower potency compared with Thyrogen. Calculations indicated that SAFA-TSH required six times the weight-based dose of Thyrogen to achieve equivalent cAMP levels, attributed to differences in molecular weight and relative bioactivity.

#### *In vivo* PK assays

**Figure 4:**
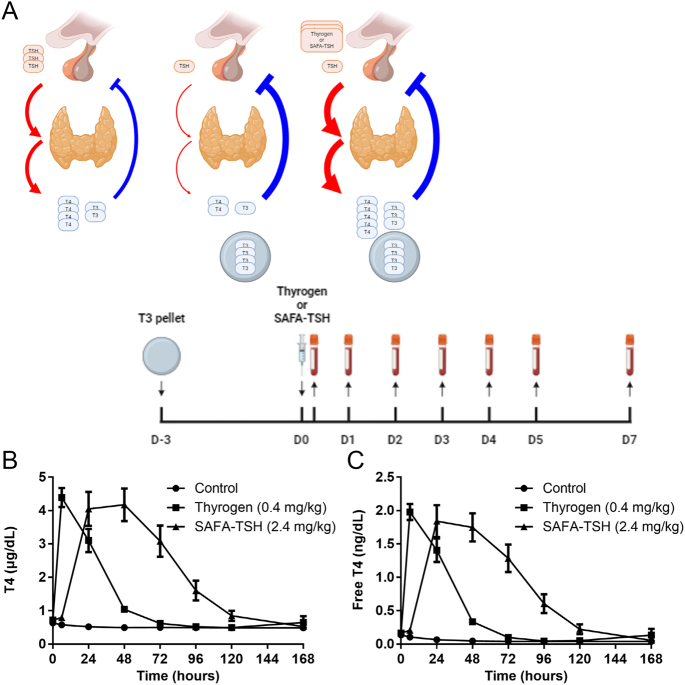
PD study results of anti-serum albumin Fab-associated (SAFA)-TSH. (A) Time course of Sprague–Dawley (SD) rat *in vivo* experiments (Created with BioRender.com), (B and C) serum thyroxine (T4) and free T4 levels at various time points after Thyrogen or SAFA-TSH injection. Line plots indicate the mean ± standard error of the mean (SEM). A full color version of this figure available at https:// doi.org/10.1530/ERC-24-0284.

The PK profiles of SAFA-TSH were evaluated following subcutaneous administration to SD rats, with Thyrogen used as a reference. Data from the PK studies are shown in Fig. 4 and summarized in Table 1. Thyrogen at 0.5 mg/kg resulted in a maximum concentration (C_max_) of 211.0 ng/mL, area under the curve (AUC)_last_ of 2,609.6 h × ng/mL and an AUC_inf_ of 2,582.3 h × ng/mL, with a biological half-life (T_1/2_) ranging from 3.6 to 4.2 h (median: 4.0 h), apparent volume of distribution (Vz/F) of 1,104.1 mL/kg, apparent clearance (CL/F) of 194.9 mL/h/kg and MRT_last_ from 6.2 to 7.1 h (median: 6.4 h).

**Table 1:** PK parameters for subcutaneous administration of recombinant Thyrogen and SAFA-TSH in SD rats.

Parameters (units)	Thyrogen	SAFA-TSH	SAFA-TSH
	0.5 mg/kg	1.5 mg/kg	4.5 mg/kg
C_max_ (ng/mL)	211.0	2,631.1	8,049.8
AUC_last_ (h × ng/mL)	2,609.6	150,219.6	468,639.7
AUC_inf_ (h × ng/mL)	2,582.3	189,546.7	558,865.2
T_max_ (h)	2	24	24
T_1/2_ (h)	4.0	38.8	30.4
Vz/F (mL/kg)	1,104.1	439.8	359.8
CL/F (mL/h/kg)	194.9	8.0	8.1
MRT_last_ (h)	6.4	44.2	43.6
AUC_extrap_ (%)	1.1	20.7	16.0

Abbreviations: PK, pharmacokinetic; SAFA, anti-serum albumin Fab-associated; TSH, thyroid-stimulating hormone; SD, Sprague–Dawley; C_max_, maximum concentration; AUC_last_, area under the curve to last measurable concentration; AUC_inf_, area under the curve to time infinity; T_max_, time to reach C_max_; T_1/2_, biological half-life; Vz/F, apparent volume of distribution; CL/F, apparent clearance; MRT_last_, mean residence time to last measurable concentration; AUC_extrap_, extrapolated area under the curve, ((AUC_inf_ − AUC_last_)/AUC_inf_) × 100.

In contrast, SAFA-TSH administered at 1.5 mg/kg showed higher values: C_max_ of 2,631.1 ng/mL, AUC_last_ of 150,219.6 h × ng/mL and AUC_inf_ of 189,546.7 h × ng/mL, with a longer T_1/2_ ranging from 33.0 to 45.0 h (median: 38.8 h), Vz/F of 439.8 mL/kg, CL/F of 8.0 mL/h/kg and MRT_last_ from 41.7 to 45.1 h (median: 44.2 h).

At a higher dose of 4.5 mg/kg, SAFA-TSH demonstrated significantly elevated parameters: C_max_ of 8,049.8 ng/mL, AUC_last_ of 468,639.7 h × ng/mL and AUC_inf_ of 558,865.2 h × ng/mL, with a T_1/2_ ranging from 26.8 to 34.0 h (median: 30.4 h), Vz/F of 359.8 mL/kg, CL/F of 8.1 mL/h/kg and MRT_last_ from 41.6 to 45.8 h (median: 43.6 h).

SAFA-TSH consistently exhibited a higher C_max_ and AUC, longer T_1/2_, smaller Vz/F, lower CL/F and longer MRT_last_ compared with Thyrogen. Increasing the SAFA-TSH dose three-fold led to a proportional rise in C_max_ (3.06 times) and AUC_last_ (3.12 times), while the T_1/2_, Vz/F, CL/F and MRT_last_ showed minimal change relative to dose escalation.

#### *In vivo* PD assays

In the PD study, 6-week-old male SD rats were implanted with a 0.5 mg T3 pellet and subsequently administered either Thyrogen (0.4 mg/kg) or SAFA-TSH (2.4 mg/kg) (Fig. 4A). Serum samples collected over several time points revealed distinct patterns in thyroid hormone levels. The control group maintained suppressed serum T4 and free T4 levels, indicating consistent thyroid function with suppressed TSH under the influence of the T3 pellet (Fig. 4B and C). In contrast, Thyrogen administration resulted in increased serum T4 and free T4 levels, peaking within 24 h post-administration and returning to baseline before day 3. SAFA-TSH administration led to a pronounced and sustained elevation in both serum T4 and free T4 levels, beginning as early as 6 h post-administration, peaking between 24 and 48 h and persisting before returning to baseline by day 7. SAFA-TSH had a significantly higher cumulative effects on T4 and free T4 levels compared with Thyrogen, as observed by a more than two-fold higher average area under the effect curve of 262.56 vs 118.89 μg × h/dL and 127.47 vs 60.75 μg × h/dL, respectively. These findings highlight SAFA-TSH’s potent and prolonged effect on thyroid hormone modulation, underscoring its potential as an advanced therapeutic option for conditions requiring precise thyroid hormone control, such as in thyroid cancer treatment strategies.

## Discussion

In this study, we successfully developed a new long-acting rhTSH using SAFA technology. Our validation through *in vitro* assays, PK studies in healthy mice and PD studies in a TSH-suppressed mouse model confirmed its biological activity. Traditional rhTSH, such as Thyrogen, is limited by its short half-life, necessitating a multidose regimen for effective treatment and diagnostic purposes (Giovanella & Duntas 2019). However, the extended duration of action provided by the long-acting rhTSH developed using SAFA technology not only has the potential to offer greater convenience for patients but also enhance therapeutic efficacy. Patients would no longer need to visit the hospital multiple times for injections, and the administration of radioactive iodine could be scheduled more flexibly, potentially leading to better stimulation of thyroid cancer cells. Due to their increased and sustained uptake, thyroid cancer cells become more avid for radioactive iodine, which is expected to enable more effective treatment with lower doses of radioactive iodine without causing discomfort to the patient (Kogai *et al.* 1997).

In previous studies, we successfully developed a long-acting recombinant human FSH (SAFA-FSH), a pituitary hormone similar to TSH (Kim *et al.* 2023). FSH and TSH share a common alpha subunit; however, their beta subunits differ (Boime & Ben-Menahem 1999). Therefore, we aimed to create SAFA-TSH v1 by only modifying the beta subunits in SAFA-FSH. However, SAFA-TSH v1 showed poor heavy-chain expression and inadequate structure formation. Therefore, in SAFA-TSH v2, we modified the linkage of the beta and alpha subunits to improve expression efficiency. The improved expression and stability of SAFA-TSH v2 show that our modifications resolved the issues with the initial construct.

The FRTL-5 cells commonly used in thyroid cell functional studies originate from rat thyroids and require a complex culture medium (Thotakura *et al.* 1991, Kogai *et al.* 1997). Although the response of Nthy-ori 3-1 cells to TSH stimulation is relatively weaker compared with primary thyroid cells, there is a study that also observes adenylate cyclase activation in Nthy-ori 3-1 cells (Piotrowska *et al.* 2011). However, Nthy-ori 3-1 cells, which are derived from human thyroid, did not show an increase in intracellular cAMP levels when exposed to Thyrogen in this study. This led to the creation of Nthy-ori 3-1_TSHR cells via lentiviral overexpression of the TSHR gene, which demonstrated measurable cAMP production with both SAFA-TSH and Thyrogen, increasing cAMP levels in these cells in a dose-dependent manner. The molecular weight of SAFA-TSH is estimated to be 71.6 kDa, but actual measurements show it to be 80.6 kDa due to glycosylation, whereas Thyrogen has a molecular weight of 28 kDa (Estrada *et al.* 2014). Even after accounting for molecular weight, SAFA-TSH requires approximately 2.1 times the molar amount of Thyrogen to achieve comparable biological activity. The higher molecular weight likely results in reduced affinity.

In this study, the PK profiles of SAFA-TSH in SD rats revealed a significantly prolonged half-life compared with Thyrogen, indicating that SAFA-TSH remains in the bloodstream longer and reduces the need for frequent dosing. In terms of PD, SAFA-TSH provides a more prolonged and significant increase in serum T4 and free T4 levels compared with Thyrogen. Its effects last longer, leading to more sustained thyroid stimulation (Kowalska *et al.* 2015). The half-life of SAFA-TSH in humans is expected to be longer than in rats due to the superior affinity and extended half-life of human serum albumin. Based on similar technology used in SAFA-IL-18BP, SAFA-TSH is anticipated to have a prolonged half-life of approximately 2 weeks in humans (Jang *et al.* 2023). In addition, the lower peak-to-trough T4 effect observed with SAFA-TSH may minimize the risk of adverse effects associated with sudden TSH level changes. The substantially increased overall exposure of SAFA-TSH could potentially enhance therapeutic efficacy by improving radioiodine uptake, although this hypothesis will need to be validated through clinical trials (Giovanella & Duntas 2019).

This development in SAFA-TSH is not the first attempt to develop a long-acting form of TSH. Previous research has demonstrated that carbohydrate-mediated polyethylene glycol (PEG) conjugation of TSH improves its pharmacological properties (Park *et al.* 2013). Their leading candidate showed a significantly longer duration of action in rats compared with rhTSH, with its extended circulation half-life more than compensating for its lower affinity. While PEGylation has successfully extended the half-lives of many therapeutics, it raises concerns due to its nonbiodegradable nature and potential for immunogenic responses (Zaman *et al.* 2019). Given that Fab fragments have demonstrated clinical safety, we are confident that SAFA technology has considerable potential as a carrier molecule for prolonging the serum half-life of therapeutic proteins (Ji *et al.* 2019, Zaman *et al.* 2019).

In addition to its potential in radioiodine therapy for DTC management, SAFA-TSH could be utilized in various other applications, although further research is needed. (Duntas & Cooper 2008). It may enhance the sensitivity of fluorodeoxyglucose positron emission tomography/computed tomography scans, which can be beneficial for more accurate diagnosis and treatment planning (Chin *et al.* 2004, Saab *et al.* 2006, Leboulleux *et al.* 2009). Furthermore, SAFA-TSH holds promise in the diagnosis of congenital hypothyroidism and could improve the efficacy of ^131^I treatment for nodular goiter (Fugazzola *et al.* 2003, Graf 2015). In addition, there is potential for SAFA-TSH to be used in the treatment of central hypothyroidism, expanding its therapeutic applications beyond current uses (Filipsson *et al.* 2008, Persani 2012, 2019).

Nevertheless, this study had limitations. First, our PK and PD studies were conducted in rats, and the extrapolation of these findings to humans must be approached with caution. Differences in the metabolism, PKs and receptor dynamics between species can impact the translation of these results to human in clinical settings. Therefore, further studies in humans are essential to fully understand the clinical implications of SAFA-TSH. Second, while the extended half-life of SAFA-TSH represents a significant advancement over traditional rhTSH formulations, clinical trials are required to investigate the potential long-term effects and safety profile of prolonged TSH exposure. Extended duration of action may have unforeseen impacts on thyroid and other physiological processes, which need to be carefully monitored. Third, although we demonstrated that SAFA-TSH has a more prolonged effect on T4 and free T4 levels compared to Thyrogen, the comparative efficacy in enhancing radioiodine uptake and improving clinical outcomes for thyroid cancer patients remains to be established. Prospective clinical trials are necessary to evaluate the effect of SAFA-TSH on clinical endpoints, such as disease control rates and patient quality of life.

In conclusion, the development of SAFA-TSH using SAFA technology marks a significant advancement in the treatment of thyroid cancer. Its extended half-life and sustained bioactivity offer clear advantages over traditional rhTSH formulations, potentially improving patient outcomes and quality of life. Future clinical trials are critical to validate these findings and to establish an optimal dosing regimen. Continued development and evaluation of SAFA-TSH holds promise for a major breakthrough in the management of thyroid cancer, underscoring the need for further research to fully realize its therapeutic potential.

## Declaration of interest

At the time of publication, Gunuk Zi, Jeongsuk An, Jinjoo Park, Jaekyu Han, Susan Chi and Sang-hoon Cha were employees of AprilBio Co., Ltd, Chuncheon, South Korea. Daham Kim, Min Jeong Kang, So Jeong Lee, Yoon Hee Cho and Eun Jig Lee declare that they have no competing interests. All authors have read and approved the final manuscript.

## Funding

This research was supported by a grant from the Korea Health Technology R & D Project through the Korea Health Industry Development Institutehttps://doi.org/10.13039/501100003710 (KHIDI), funded by the Ministry of Health and Welfarehttps://doi.org/10.13039/501100003625, Republic of Korea (grant number: HR18C0012).
